# Malignant Gastrointestinal Neuroectodermal Tumor (GNET) with Prolonged Disease-Free Survival after Platinum-Based Chemotherapy

**DOI:** 10.1155/2020/8880202

**Published:** 2020-06-30

**Authors:** Daulath Singh, Mohammed K. Atieh, Mark A. Russell, Muaiad Kittaneh

**Affiliations:** ^1^Department of Medicine-Oncology, Loyola University Chicago, 2160 S. First Ave, Maywood, IL 60153, USA; ^2^Department of Pathology, Loyola University Chicago, 2160 S. First Ave, Maywood, IL 60153, USA

## Abstract

Malignant gastrointestinal neuroectodermal tumor (GNET) is a rare disease with a handful of cases described in literature. GNET has only become a well-known/widely accepted entity in the recent years, but it is still not listed in the database of rare diseases. Due to the rarity of disease, there are no guidelines on standard therapeutic approaches in the adjuvant or metastatic setting. Here, we describe a unique case of GNET with a 7-year disease-free survival following adjuvant cisplatin and etoposide chemotherapy. This is the longest disease-free survival that has ever been described in literature and may support using this combination in a larger cohort of patients in the context of a global clinical trial. We will also review the histopathologic features of GNET and potential therapeutic options in the metastatic setting.

## 1. Introduction

Malignant gastrointestinal neuroectodermal tumor (GNET) is a rare primitive mesenchymal neoplasm of the gut. It was previously known as clear cell sarcoma-like tumor of the GI tract (CCSLTGT) [1]. In the recent years, it has been recognized as a separate entity with specific histologic, immunohistochemistry, and molecular features [2]. GNET carries a poor prognosis, and systemic therapeutic options are very limited with lack of guidelines or management recommendation. Reported literature lists cytotoxic chemotherapy and various targeted agents that have been used for the treatment of this entity (with limited benefit). Here, we report a case of GNET that was treated with surgical resection and adjuvant chemotherapy (of cisplatin and etoposide) followed by disease-free interval of 7 years prior to recurrence. The recurrence was very aggressive, and patient expired despite use of multiple lines of treatment. To our knowledge, this case represents the first literature report with the longest disease-free survival following definitive surgical resection followed by adjuvant chemotherapy of a GNET.

## 2. Case Presentation

A 61-year-old African American male presented to a local community hospital in 2010 with abdominal pain. A CT of the abdomen revealed an obstructing mass in the right colon, and the patient underwent a right hemicolectomy. Pathologic evaluation at that time was felt to be consistent with pT3N2 poorly differentiated neuroendocrine carcinoma with lymphovascular/venous invasion (LVI), and the patient received 4 cycles of adjuvant chemotherapy with cisplatin and etoposide (VP-16). He was subsequently followed by clinical and imaging surveillance with no evidence of recurrent disease until 2017 when he presented with a 4-week history of postprandial abdominal discomfort. His physical exam was unremarkable.

**Figure 1:**
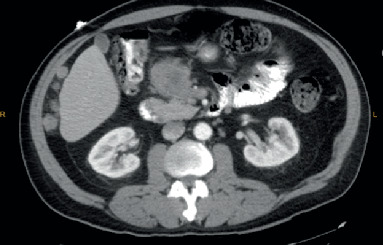
Showing the peripancreatic mass.

A contrast-enhanced Computed Tomography (CT) of the abdomen and pelvis (AP) showed infiltrative appearing heterogeneous enhancing mass within the mesenteric fat anterior to the pancreatic head measuring 4.9 × 5.5 cm and few additional foci of peritoneal implants (Figure 1). Laboratory evaluation showed an elevated level of chromogranin A at 139 (normal 0-95 ng/ml) and gastrin level at 161 (normal ≤ 100 pg/ml). Due to previous reported history of neuroendocrine tumor, a Gallium 68 DOTATATE Scan was performed but showed no evidence of somatostatin receptor overexpression. An endoscopic ultrasonography (EUS) showed an irregular hypoechoic 4.6 cm mass in the peripancreatic region adjacent to the pancreatic head without involvement of the pancreatic parenchyma and a 10 × 9 mm malignant-appearing lymph node in the peripancreatic region.

A fine needle aspiration biopsy of the peripancreatic mass showed clusters of molding tumor cells and single tumor cells with enlarged, hyperchromatic nuclei and finely granular chromatin. No significant mitotic activity was observed. Although the cell block sections were very low cellular, immunohistochemical stains were attempted, and the few cells present were negative for all markers applied (synaptophysin, chromogranin, and keratin AE1/AE3). Given the history of poorly differentiated neuroendocrine carcinoma, the findings were felt to be consistent with the patient's reported past medical history.

The patient was initially treated with systemic chemotherapy utilizing capecitabine 1500 mg/m^2^ po BID D1-14 and temozolomide 150 mg/m^2^ D10-14 every 28 days [2]. Imaging studies after 4 cycles of chemotherapy showed interval progression of the disease with development of numerous liver lesions and increased size of peritoneal masses.

**Figure 2:**
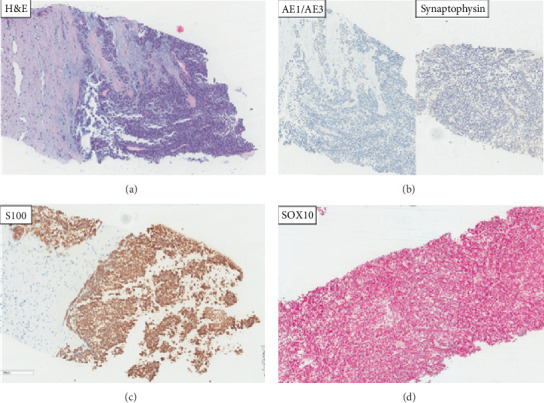
H&E (a) shows loosely discohesive epithelioid cells with eosinophilic to clear cytoplasm, round to ovoid nuclei, and prominent nucleoli with a solid growth pattern. Keratin AE1/AE3 and synaptophysin IHC negative (b). S100 (c) and SOX10 (d) IHC diffusely positive. Red chromogen IHC showing positive red staining in the nuclei.

The patient was subsequently started on second-line therapy with everolimus 10 mg daily. He remained on everolimus for 5 months achieving a mixed response as the best response to therapy. He had new and enlarging hepatic lesions and new peritoneal lesions, as well as stable to decreased size of the several hepatic lesions and resolved peritoneal lesions. A core needle biopsy of the liver mass showed areas of tumor exhibiting a nested/trabecular and focally solid growth pattern. The tumor cells were composed of loosely discohesive, bland, neoplastic epithelioid-appearing cells with round to oval, mildly irregular hyperchromatic nuclei, and moderate to high nuclear to cytoplasmic ratios (Figure 2). Immunohistochemical (IHC) staining showed the tumor cells were diffusely positive for S100, SOX-10, and Vimentin with focal positivity for CD56. Beta-catenin IHC showed diffused cytoplasmic and nuclear positivity. IHC was negative for pancytokeratin (AE1/AE3), CK 8/18, EMA, CD99, HMB-45, Mart-1, NSE, p63, CD117/CKIT, CDX2, TTF-1, SMA, and synaptophysin. Ki-67 proliferation index was 20%. The tumor in the current liver biopsy and the prior colon resection (from 2010) specimen was compared and exhibited similar morphologic appearance. In addition, IHC for SOX-10, S100, and pancytokeratin (AE1/AE3) were also performed and examined on a selected tissue block from the prior colon tumor specimen showing a similar staining pattern (positive staining for S100 and SOX-10 and negative staining for pancytokeratin (AE1/AE3)). FISH analysis showed rearrangement in the EWSR1 (22q12) locus in 87% of the interphase cells analyzed, with subsequent studies detecting a fusion of the CREB1 (2q33) and the EWSR1 loci in 90% of the cells (specifically, positive for t(2;22)(q33;q12)); no fusion of the ATF1 (12q13.13) and EWSR1 loci was present. Overall, the morphology, IHC pattern, and the presence of EWSR1 molecular FISH abnormality were consistent with a diagnosis of gastrointestinal neuroectodermal tumor (GNET), previously known as clear cell sarcoma-like tumor of the GI tract (CCSLGT). GNET is a rare tumor that has a specific immunohistochemical pattern-positive staining for S100, SOX10, and Vimentin, while showing negative staining for epithelial (cytokeratins, EMA) and melanocytic (HMB-45, Mart-1) markers. It also demonstrates two characteristic molecular FISH abnormalities described as EWSR1-ATF1 and EWSR1-CREB1 fusions [3].

Next generation sequencing using FOUNDATION ONE® CDX showed the tumor to be microsatellite (MS) status stable with low tumor mutational burden (TMB) of 3 mutations/Mb and confirmed the presence of EWSR1-CREB1 fusion.

A trial of multityrosine kinase therapy, pazopanib 800 mg daily, was pursued, but the patient developed a progressive disease with refractory hiccups due to enlarging mesenteric tumors causing irritation from a mass effect on the stomach and diaphragm after one month of therapy.

A trail of different tyrosine kinase inhibitor was used, and the patient received sunitinib 37.5 mg daily. The patient achieved a mixed radiologic response represented by improvement in mesenteric implants, but interim increase in size and number of hepatic metastasis. He remained on sunitinib for 3 months. Best supportive care was subsequently initiated, and the patient expired within a few weeks.

## 3. Discussion

Malignant gastrointestinal neuroectodermal tumors (GNETs) are rare primitive mesenchymal neoplasms of the tubular gut previously considered to be gastrointestinal manifestations of soft tissue clear cell sarcomas (CCS) [1]. GNETs were previously known as CCSLTGT (with osteoclast-like giant cells) given their overlapping histological, immunophenotypical, and molecular features with soft tissue clear cell sarcomas. GNETs and CCS share a recurrent balanced translocation involving EWSR1 on chromosome 22 and one of either partner: cAMP-dependent activating transcription factor 1 (ATF1) on chromosome 12 or less commonly, cAMP-responsive element-binding protein 1 (CREB1) located on chromosome 2. The resulting chimeric fusion proteins are postulated to activate downstream genes such as microphthalmia transcription factor 1 (MiTF1) which drive melanocytic phenotypic differentiation and survival. The absence of a melanocytic immunophenotype in GNETs (despite sharing these molecular translocations) suggests additional molecular alterations which may be unique to, and further define, GNETs. GNETs are postulated to arise from primitive neural crest progenitor cells of the gastrointestinal tract autonomic nervous system. The prognosis appears poor, although published data with follow-up information are scant, given the rarity of this disease [1].

GNET was first described in 2003 by Zambrano et al. who reported 6 cases as malignant mesenchymal neoplasm of gastrointestinal tract that were characterized by the presence of osteoclast-like multinucleated giant cells that expressed S100 protein, but staining for CD117 and melanocytic markers was negative by immunohistochemistry. GNET tends to occur mainly in young to middle-aged adults. Most of the cases have been described in the small intestine in addition to the stomach and the colon. Immunohistochemically, the neoplastic cells are characterized by strong and diffuse staining for S100 protein. They also stain with SOX10. Variable reactivity is described with CD56, synaptophysin, and neuron-specific enolase (NSE). All cases reported were uniformly negative for HMB-45, Melan-A, tyrosinase, and MiTF. They also did not express GIST markers (CD117, DOG-1, and CD34). Alyousef and his team reported 3 cases of CCS of gastrointestinal tract and claimed to be the first to describe a recurrent translocation of EWS (22q12) and CREB1 (2q32.3) resulting in EWS-CREB1 fusion [4].

IHC profile is indicative of neural differentiation as evident by positive staining with S100 (100%), SOX10 (100%), CD56 (70%), synaptophysin (56%), NB84 (50%), NSE (45%), and neurofilament (14%) [3]. They stain negative for melanocytic markers like HMB45, Melan A, and tyrosinase. Ultrastructurally, they lack melanocytic differentiation and show features of neural differentiation, including multiple interdigitating cell processes containing dense core granules and clear vesicles resembling synaptic bulbs. Stockman et al. [3] suggested origin of these tumors from an autonomic nervous system-related primitive cell of neural crest derivation that shows a neural line of differentiation with a complete absence of melanocytic features.

GNET is a rare aggressive neoplasm with a high rate of local recurrence. It is often metastatic at presentation and associated with high mortality. EWSR1-CREB1 activates the melanocyte transcription factor MITF, which in turn activates transcription of c-MET, an oncogenic receptor tyrosine kinase that was recently shown to be activated in clear cell sarcoma and GNET [5].

Other tumors that exhibit EWSR1 gene rearrangements are Ewing's sarcoma, angiomatoid fibrous histiocytoma, primary pulmonary myxoid sarcoma, and hyalinizing clear cell carcinoma of the salivary gland [6].

The differential diagnosis for GNET includes GIST, monophasic synovial sarcoma, primary or metastatic malignant melanoma, and clear cell sarcoma of tendons and aponeuroses (CCSTA). Distinguishing GNET from other sarcomas of the gastrointestinal tract, especially GIST, is important because of their different pathogenesis and response to treatment [3].

Thirteen cases of CCS-GIT and 58 of GNET were described in a more recent study. CCS-GIT occurred more commonly in males (84.6% vs. 46.6%, *p* = 0.01) and in an older age group (median 57 vs. 33 years *p* < 0.01). There was no significant difference in their location in the gastrointestinal tract, median tumor size, and proportion of cases with an EWSR1-ATF1 vs. EWSR1-CREB1 fusion. Median survival for CCS-GIT was 13.5 months and for GNET, 9.5 months (p =0.78). CCS-GIT and GNET are high-grade tumors for which conventional chemotherapy and radiotherapy presently appear to have little to offer [7].

Management options for clear cell sarcoma (CCS) and CCSLGT include surgical resection with regional lymph node dissection, radiotherapy, and chemotherapeutic regimens that include ifosfamide and doxorubicin [8]. Crizotinib and pazopanib were reported to offer a durable near complete response of about 1.5 years in one case report [8].

Another study included 19 patients with GNET with a mean tumor size of 4.2 cm. Within a mean follow-up of 29.7 months (range: 3 to 63 months), 2/15 (13.3%) patients died of disease, 5 (33.3%) were alive with disease, and 8 (53.3%) had no evidence of disease. The common management was a radical excision of the tumor with wide lymphadenectomy followed by close monitoring for local recurrence and metastasis. Clinically, there were 3 patients treated with apatinib or anlotinib in these series who experienced preliminary clinical benefit (1 partial response and 1 stable disease to apatinib, 1 partial response to apatinib), indicating that apatinib and anlotinib might be reasonable options for the treatment of advanced GNET and could prolong patient survival [9]. Another study reported a case of a 30-year-old female with distal ileum GNET s/p resection and adjuvant chemotherapy with ifosfamide and epirubicin. She was alive at the time of reporting (6 months post treatment) [10]. Given the rarity of this disease, there are currently no standard chemotherapeutic or targeted therapeutic options for this disease and literature is only available in the form of case reports or reviews. GNET is a rare disease with limited opportunity to study in randomized clinical trials to generate level one evidence on how best to treat this disease.

This case report highlights several important clinical and pathologic considerations. The patient was 1first diagnosed with pT3N2 poorly differentiated neuroendocrine carcinoma and treated with 4 cycles of adjuvant chemotherapy utilizing etoposide (VP-16) and cisplatin. He developed recurrent disease after 7 years when he was referred to our academic center. Careful review of the pathology revealed that the primary disease was gastrointestinal neuroectodermal tumor (GNET) which was confirmed by molecular testing and FISH. Diagnosis of gastrointestinal neuroectodermal tumor requires careful evaluation of morphologic, IHC, and molecular features. It is important to note that this patient had elevated chromogranin A and gastrin which are usually elevated in neuroendocrine neoplasms and may potentially create a diagnostic ambiguity for the treating clinicians. To our knowledge, this is the first case with the longest disease-free survival of 7 years (likely due to platinum-based chemotherapy and localized nature of the neoplasm) described in literature following resection and adjuvant chemotherapy with cisplatin and etoposide. There are currently no standard therapies to treat GNET in the adjuvant or metastatic setting. This case report is a hypothesis generating that etoposide (VP-16) and cisplatin may be an active regimen for GNET. Conducting traditional randomized clinical trials may not be feasible for rare diseases. Evaluating real-world data may be helpful in exploring any evidence to support the use of etoposide (VP-16) and cisplatin in the treatment of GNET. In the metastatic setting, this patient progressed on capecitabine/temozolomide combination and on pazopanib, but he was able to attain a mixed response with everolimus (for about 5 months) and with sunitinib (for ~3 months).

In conclusion, GNET is a rare mesenchymal malignancy with poor prognosis and occurs mainly in young and middle-aged adults. GNET should be suspected in cases of a gastrointestinal tract tumor comprising epithelioid cells arranged in various patterns and/or sheets of spindle cell tumor cells. Positive S100 and SOX10 expression and negative epithelioid and melanocytic marker expression on immunostaining will be helpful in arriving at the diagnosis, and molecular detection of involvement of EWSR1 chromosomal rearrangement is recommended to confirm the diagnosis of GNET. Further studies are needed to determine the best therapeutic options for this disease.

## Data Availability

This is a single-patient case report and data accessed through EMR.
